# Peroxisome Proliferator-Activated Receptor-Gamma Reduces ER Stress and Inflammation via Targeting NGBR Expression

**DOI:** 10.3389/fphar.2021.817784

**Published:** 2022-01-17

**Authors:** Jialing Ma, Peng Zeng, Lipei Liu, Mengmeng Zhu, Juan Zheng, Chengyi Wang, Xiaokang Zhao, Wenquan Hu, Xiaoxiao Yang, Yajun Duan, Jihong Han, Qing R. Miao, Yuanli Chen

**Affiliations:** ^1^ Key Laboratory of Major Metabolic Diseases and Nutritional Regulation of Anhui Department of Education, College of Food and Biological Engineering, Hefei University of Technology, Hefei, China; ^2^ College of Life Sciences, State Key Laboratory of Medicinal Chemical Biology, Key Laboratory of Bioactive Materials of Ministry of Education, Nankai University, Tianjin, China; ^3^ Centre for Lipid Research & Key Laboratory of Molecular Biology for Infectious Diseases (Ministry of Education), Chongqing Medical University, Chongqing, China; ^4^ Diabetes and Obesity Research Center, New York University Long Island School of Medicine, New York, NY, United States

**Keywords:** PPARγ, rosiglitazone, NGBR, ER stress, inflammation

## Abstract

Increased Nogo-B receptor (NGBR) expression in the liver improves insulin sensitivity by reducing endoplasmic reticulum stress (ER stress) and activating the AMPK pathway, although it remains elusive the mechanisms by which NGBR is induced. In this study, we found that PPARγ ligands (rosiglitazone or pioglitazone) increased NGBR expression in hepatic cells and HUVECs. Furthermore, promoter analysis defined two PPREs (PPARγ-responsive elements) in the promoter region of NGBR, which was further confirmed by the ChIP assay. *In vivo*, using liver-specific PPARγ deficient (PPARγ^LKO^) mice, we identified the key role of PPARγ expression in pioglitazone-induced NGBR expression. Meanwhile, the basal level of ER stress and inflammation was slightly increased by NGBR knockdown. However, the inhibitory effect of rosiglitazone on inflammation was abolished while rosiglitazone-inhibited ER stress was weakened by NGBR knockdown. Taken together, these findings show that NGBR is a previously unrecognized target of PPARγ activation and plays an essential role in PPARγ-reduced ER stress and inflammation.

## Introduction

The endoplasmic reticulum (ER) is an essential multifunctional organelle, which is the primary place for protein synthesis, folding, and assembly, as well as degradation of misfolded or damaged proteins. When ER homeostasis is disrupted, misfolded proteins gather in the ER and subsequently activate the unfolded protein response (UPR) (Walter and Ron, 2011). After activation, UPR will reconstruct cellular transcription and translation, as well as degradation pathways to resolve defects in protein folding. These effects are accomplished by activating three transmembrane ER proteins, namely protein kinase RNA-dependent ER kinase (PERK), inositol-requiring enzyme 1 (IRE-1), and activating transcription factor 6 (ATF6) (Hetz, 2012). ER stress links to many inflammatory and stress signaling, including the activation of the nuclear factor κB (NF-κB) pathway (Kharroubi et al., 2004). Both inflammation and ER stress are short-term adaptive systems that are indispensable for the function and survival of the organism (Hotamisligil, 2010). However, they are detrimental when the inflammation and ER stress are chronically engaged (Hotamisligil, 2010; Fu et al., 2012). ER stress and chronic inflammation are related to many diseases, including diabetes, obesity, neurodegenerative and neuromuscular inflammatory diseases (Xue et al., 2016). ER stress can strengthen many stress and inflammatory signal pathways, aggravate metabolic disorders, and lead to obesity, insulin resistance, NAFLD, and dyslipidemia (Wang and Kaufman, 2012).

On the one hand, Nogo-B receptor (NGBR) was identified as a receptor for Nogo-B in human umbilical vein endothelial cells (HUVECs). Restraining NGBR expression can eliminates Nogo-B-mediated endothelial cell (EC) migration, attenuates vascular endothelial growth factor (VEGF)-stimulated phosphorylation of Protein kinase B (Akt) and HUVECs chemotaxis and morphogenesis (Miao et al., 2006). On the other hand, NGBR can independently affect nervous system regeneration. Moreover, NGBR can promote Niemann-Pick type C2 protein (NPC2)-dependent cholesterol trafficking and AMPKα-liver X receptor (LXR)-dependent free fatty acids (FFA) and triglycerides (TG) metabolism (Hu et al., 2016; Zhang et al., 2018). NGBR may promote or inhibit the occurrence and progression of tumor, which depends on the types of tumors (Wu et al., 2018). Our previous studies demonstrate that overexpression of NGBR improves glucosamine hydrochloride (Glcn)-induced ER stress. Likewise, overexpression of NGBR in the liver reduced ER stress and increased insulin sensitivity in high-fat diet (HFD)/streptozotocin (STZ)-treated mice tissues, suggesting that hepatic NGBR may be necessary to maintain insulin sensitivity and prevent type 2 diabetes (T2D) (Chen et al., 2021).

Three subtypes of peroxisomal proliferator-activated receptors (PPARs) have been identified: PPARα, β and γ, which are all ligand-activated nuclear transcriptional factors. PPAR and retinoid X receptor (RXR) can form a heterodimer to bind to a DNA recognition motif named PPAR response element (PPRE), which consists of a direct repeat separated by one nucleotide (also known as DR1) with a consensus sequence of AGGTGA-X-AGGTCA. Synthetic PPARγ agonists, including rosiglitazone and pioglitazone, effectively enhance insulin sensitivity and are currently used as drugs for T2D (Huang et al., 2021). Studies have demonstrated that ER stress and inflammatory responses are downregulated after treatment with various PPARγ agonists (Ricote et al., 1998; Straus and Glass, 2007; Yoshiuchi et al., 2009) across multiple cell types and tissues. However, the mechanisms underlying PPARγ-inhibited ER stress and inflammation are not fully understood.

Considering the functions of NGBR in EC and liver, which all contribute to insulin sensitivity and inflammation, we determined the effect of PPARγ activation on NGBR expression and function *in vitro* with EC and hepatocytes and *in vivo*. We proved that NGBR was a previously unrecognized target of PPARγ. Besides, we also tried to reveal that PPARγ ligand-alleviated inflammation and ER stress were related to NGBR expression.

## Materials and Methods

### Reagents

Rosiglitazone, pioglitazone and GW9662 were purchased from Cayman Chemical (Ann Arbor, MI, United States). LPS and tunicamycin were purchased from Sigma-Aldrich (St. Louis, MO, United States). Rabbit anti-NGBR, β-actin, and GAPDH polyclonal antibodies were purchased from Abcam (Cambridge, MA, United States). Rabbit anti-IL-1β and IL-6 polyclonal antibodies were purchased from ABclonal (Wuhan, China). Rabbit anti-PPARγ and AKT polyclonal antibodies and mouse anti-TNF-α and phospho-AKT (*p*-AKT, Ser473) monoclonal antibodies were purchased from Proteintech Group (Chicago, IL, United States). Rabbit anti-CHOP, BIP, ATF6, phospho-IRE1α (p-IRE1α, Ser724) and phospho-PERK (p-PERK, Thr982) polyclonal antibodies were purchased from Affinity Biosciences (Cincinnati, OH, United States). Silencer siRNA Construction Kit was purchased from Life Technologies (New York, United States). Transfection reagent Lipofectamine RNAiMAX and Lipofectamine 2000 were purchased from Thermo Fisher Scientific (Waltham, MA, United States).

### Cell Culture

HUVEC (human umbilical vein endothelial cell), HepG2 (human hepatoma cell line), and 293T (human embryonic kidney cell) were purchased from ATCC (Manassas, VA, United States) and cultured in DMEM containing 10% fetal bovine serum (FBS), 50 μg/ml of Pen-Strep and 2 mM glutamine. Cells received treatment at 80–90% confluence. Mouse primary hepatocytes were isolated from PPARγ^flox/flox^ and liver-specific PPARγ knockout (PPARγ^LKO^) mice by a collagenase perfusion method as described (Zhang et al., 2018).

### Western Blotting

After treatment, total cellular proteins were extracted from primary hepatocytes, HUVEC, HepG2 cells, or a piece of mouse liver, then the expression of PPARγ, NGBR, AKT, *p*-AKT, CHOP, BIP, IL-1β, TNF-α, NF-κB, ATF6, p-PERK, p-IRE1α protein was detected by Western blotting (Chen et al., 2012). After capturing the images, all bands from three repeated experiments were quantified with statistical analysis by two technicians who were blinded to the treatments.

### SiRNA Transfection

HepG2 cells or HUVECs were divided into a six-well culture plate. After reaching 30–40% confluence, cells were transfected with negative control siRNA (si-NC) or NGBR siRNA (siNGBR) using Lipofectamine RNAiMAX (Duan et al., 2012). After 24 h transfection, the cells were treated with rosiglitazone (10 μM) for 12 h, then treated with LPS (100 ng/ml) with or without rosiglitazone for another 12 h. The corresponding protein expression was determined.

### Real-Time Quantitative RT-PCR (qPCR)

The total RNApure reagent (Zomanbio, Beijing, China) was used to extract total RNA from cells or a piece of the liver. Then, 1 μg total RNA from each sample was reverse-transcribed into cDNA using a reverse transcription kit, followed by quantitative PCR according to the manufacturer’s instructions for SYBR Green Master Mix (Bio-Rad) and the primers were listed in Table 1. GAPDH or β-actin were used for normalization.

**TABLE 1:** The sequences of primers for qPCR analysis.

Gene	Forward	Reverse
h-NGBR	AGC​CTC​GTG​GTG​TGG​TGT​A	GCC​CAG​AAG​TTC​TTG​CTG​TT
h-GAPDH	GGT​GGT​CTC​CTC​TGA​CTT​CAA​CA	GTT​GCT​GTA​GCC​AAA​TTC​GTT
h-PPARγ	TCA​AAG​GAG​TGG​GAG​TGG​TC	CAA​GGC​CAT​TTT​CTC​AAA​CG
h-CHOP	GGA​AAC​AGA​GTG​GTC​ATT​CCC	CTG​CTT​GAG​CCG​TTC​ATT​CTC
h-BIP	TTG​ACT​CCG​ACC​TTC​ACC​TTC​C	TTT​CAC​AGT​GGC​CAA​GAG​TC
h-TNF-α	CGT​CGT​AGC​AAA​CCA​CCA​AG	TTG​AAG​AGA​ACC​TGG​GAG​TAG​ACA
h-IL-1β	GAC​CTT​CCA​GGA​TGA​GGA​CA	AGC​TCA​TAT​GGG​TCC​GAC​AG
h-NF-κB	GCT​CTT​ACT​GAC​TGG​CAT​GAG	CGC​AGC​TCT​AGG​AGC​ATG​TG
m-NGBR	GAG​GAA​GCC​CAC​AGA​TCT​GGA​TGT​A	TCT​GAT​TTG​CCA​GGG​AAG​AAA​GCC
m-GAPDH	ACC​CAG​AAG​ACT​GTG​GAT​GG	ACA​CAT​TGG​GGG​TAG​GAA​CA

### NGBR Luciferase Reporter Assay

PPARγ overexpression plasmid was constructed as described (Duan et al., 2012). Human NGBR promoter (from −983 to +167, pNGBR) sequence was amplified using PCR technic with genomic DNA (extracted from HepG2) and the following primers: forward, 5′-TGC​ACT​CGA​GGA​TGA​TAG​AGG​ATG​TA-3′ and reverse, 5′-TGC​CAA​GCT​TAC​TCT​TGT​GGC​CCT​C-3’. After confirming the sequence, the PCR product was double digested and ligated into the pGL4 luciferase reporter vector (Promega). The pNGBR plasmid with PPRE deletion (pNGBR-PPRE1-del, pNGBR-PPRE2-del, pNGBR-PPREs-del) was designed and constructed using the Phusion site-directed mutagenesis kit (New England Biolabs, Ipswich, MA).

293T cells were seeded into 48-well plate. After reaching ∼90% confluence, the cells were transfected with the corresponding human pNGBR plasmid and *Renilla* plasmid (20:1) using Lipofectamine™ 3000 (Invitrogen, Waltham, MA, United States). After 12 h, the cells were received the indicated treatment for 24 h in serum-free medium. Then, the cells were harvested and used to determine *Firefly* and *Renilla* luciferase activity with the Dual-Luciferase Reporter Assay System (Promega, Madison, WI, United States) as described (Yu et al., 2016).

### Chromatin Immunoprecipitation (ChIP) Assay

After the indicated treatment, the HUVECs were cross-linked in 1% formaldehyde solution, then cellular nuclear protein was extracted using the commercial kit (Keygen Biotech, Nanjing, China). ChIP assay was performed as previously described (Yu et al., 2020). The sequences of primers used in PCR as follows: PPRE1 forward, 5′-ATC​GCA​TTG​AAG​ACC​ACG​TGT​G-3′; reverse, 5′-GCC​CTC​AGA​GAC​ACC​CCC​TCT​C-3′; PPRE2 forward, 5′-GAT​GAT​AGA​GGA​TGT​AGC​ATG-3′, reverse, 5′-CAA​GAC​TTT​GAC​CAA​CCG​CCG​T-3’. The results were quantified with ImageJ software (National Institutes of Health, Bethesda, MD, United States).

### *In vivo* Studies With Animals

All the animal studies were approved by the Ethics Committee of the Hefei University of Technology and conformed to the Guide for the Care and Use of Laboratory Animals published by NIH. Animal studies are reported in compliance with the ARRIVE guidelines (Kilkenny et al., 2010; Percie Du Sert et al., 2020).

Hepatic-specific PPARγ knockout mice with C57BL/6J background (PPARγ^LKO^) and the littermate control mice (PPARγ^flox/flox^) was acquired as described previously (Yang et al., 2015).

PPARγ^LKO^ mice and PPARγ^flox/flox^ mice aged ∼8 weeks were divided into four groups (*n* = 6). Then the mice were treated with pioglitazone (30 mg/kg bodyweight) or corn oil i.g. for 5 days as previously described (Yu et al., 2020). Then, all mice were euthanized by i.p injection of an overdose of pentobarbital (500 mg/kg). Liver samples were collected.

### Data Analysis

We repeated all the experiments at least three times, with representative results presented. Values are presented as means ± SEM or means ± SD. The Shapiro Wilk method was used to analyze the normal distribution of the original data, and then Levene test was performed for the homogeneity of the square difference with SPSS software. GraphPad Prism 7 was used for statistical data analysis. Student’s t-test was used for two groups and one-way ANOVA followed by Bartlett’s test was used for more than two groups. The difference was considered significant at *p < 0.05, **p < 0.01 and ***p < 0.001.

## Results

### PPARγ Activation Induces NGBR Expression

**FIGURE 1:**
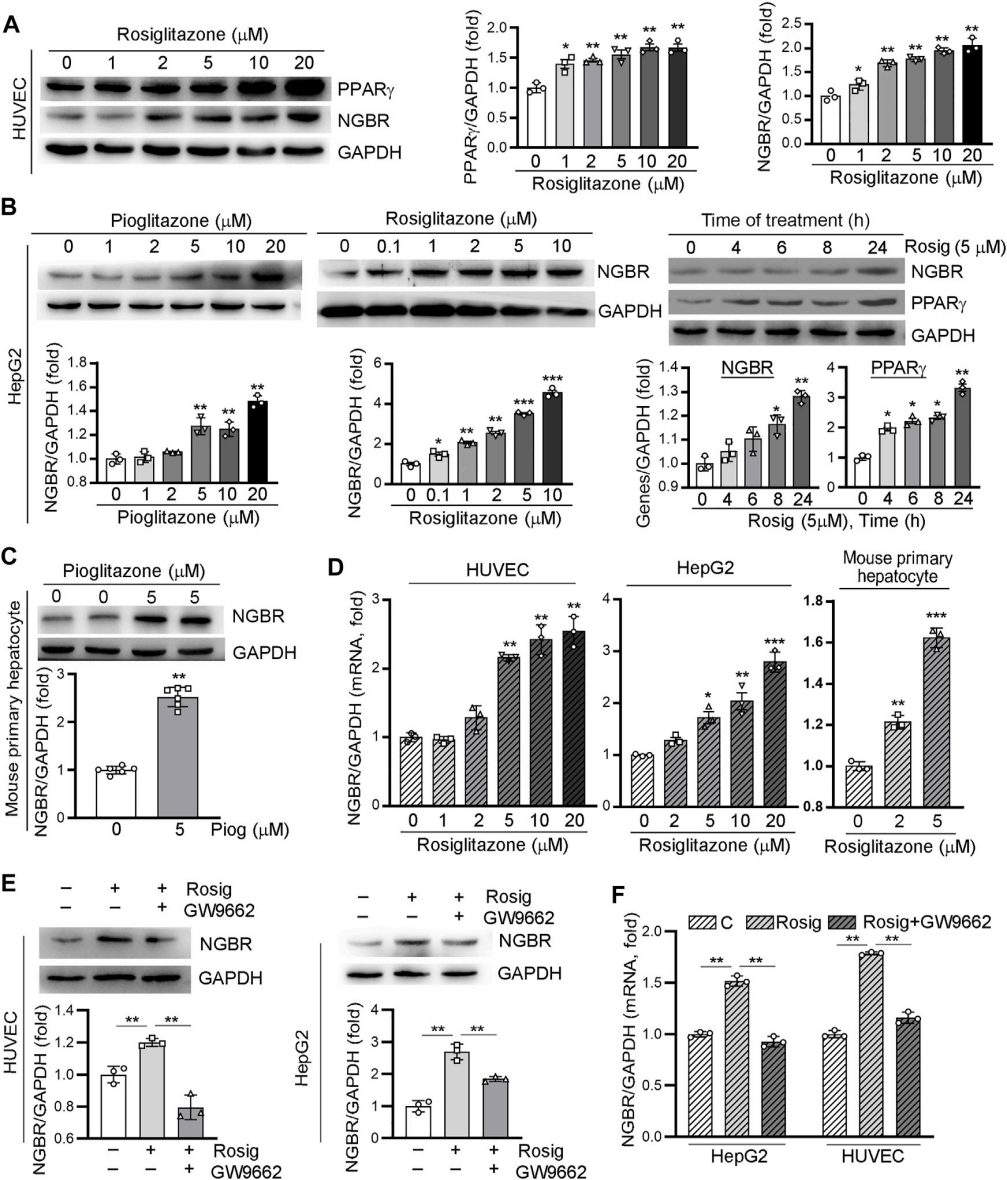
Activation of PPARγ by ligand induces NGBR expression. **(A–D)** HUVECs, HepG2 cells and mouse primary hepatocytes were treated with rosiglitazone or pioglitazone at the indicated concentrations for 24 h or pioglitazone (5 μM) for the indicated times. **(E,F)** HUVECs or HepG2 cells were treated with rosiglitazone (10 μM) with or without GW9662 (10 μM) for 24 h. Levels of PPARγ and NGBR proteins and mRNA was determined by Western blot **(A-C,E)** or qPCR **(D,F)**, respectively. Values were expressed as means ± SD, *p < 0.05; **p < 0.01; ***p < 0.001 vs control (*n* = 3).

To determine whether PPARγ activation can influence NGBR expression, we firstly treated HUVEC cells with a synthetic PPARγ ligand, rosiglitazone, at different concentrations for 24 h. The PPARγ and NGBR protein expression levels were detected by Western blot. As expected, PPARγ was increased by rosiglitazone treatment. In line with increased PPARγ expression, NGBR protein expression was increased by rosiglitazone in a concentration-dependent manner (Figure 1A). Meanwhile, we observed similar results in HepG2 cells and mouse primary hepatocytes with PPARγ ligand treatment (Figures 1B,C). The induction of NGBR expression by rosiglitazone occurred quickly after treatment, and the maximal induction was observed at 24 h after treatment (right panel, Figure 1B). With the increase in protein levels, rosiglitazone also increased NGBR mRNA expression (Figure 1D), suggesting PPARγ activates the expression of NGBR at the transcriptional level. Moreover, we found that rosiglitazone-stimulated NGBR protein and mRNA expression in HUVECs and HepG2 cells were significantly reduced by the PPARγ-specific blocker GW9662 (Figures 1E,F), indicating these effects on NGBR expression were mediated by the PPARγ signaling pathway.

**FIGURE 2:**
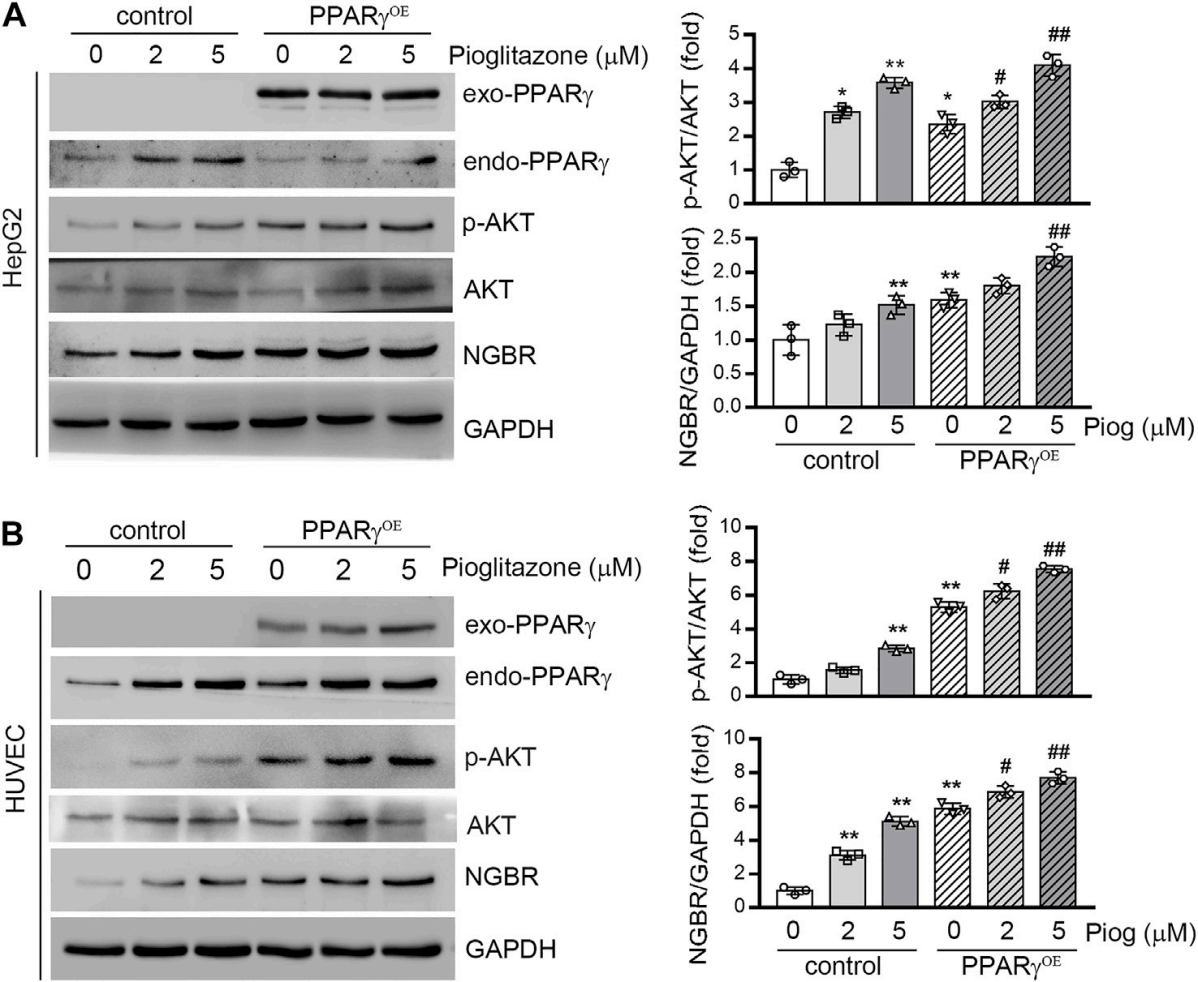
Overexpression of PPARγ increases NGBR expression. HepG2 cells **(A)** or HUVEC **(B)** were transfected with the control vector (pEGFP-C2) or PPARγ overexpression vector (pEGFP-PPARγ, PPARγ^OE^) for 24 h. The cells were then treated with pioglitazone at the indicated concentrations for 24 h. Expression of NGBR, endo-PPARγ and exo-PPARγ, *p*-AKT and AKT was determined by Western blot. Values were expressed as means ± SD, *p < 0.05; **p < 0.01 vs control group (*n* = 3); #*p* < 0.05; ##*p* < 0.01 vs PPARγ^OE^ (lone group) (*n* = 3).

To further confirm that PPARγ induced NGBR expression, we transfected HepG2 cells and HUVEC with a PPARγ overexpression vector. As shown in Figure 2, PPARγ ligand-increased NGBR expression was further enhanced by PPARγ overexpression. Similar to the expression of NGBR, pioglitazone and PPARγ overexpression enhanced AKT phosphorylation synergistically, suggesting that PPARγ may improve insulin sensitivity via regulating NGBR expression.

### Identification of PPRE in NGBR Promoter

**FIGURE 3:**
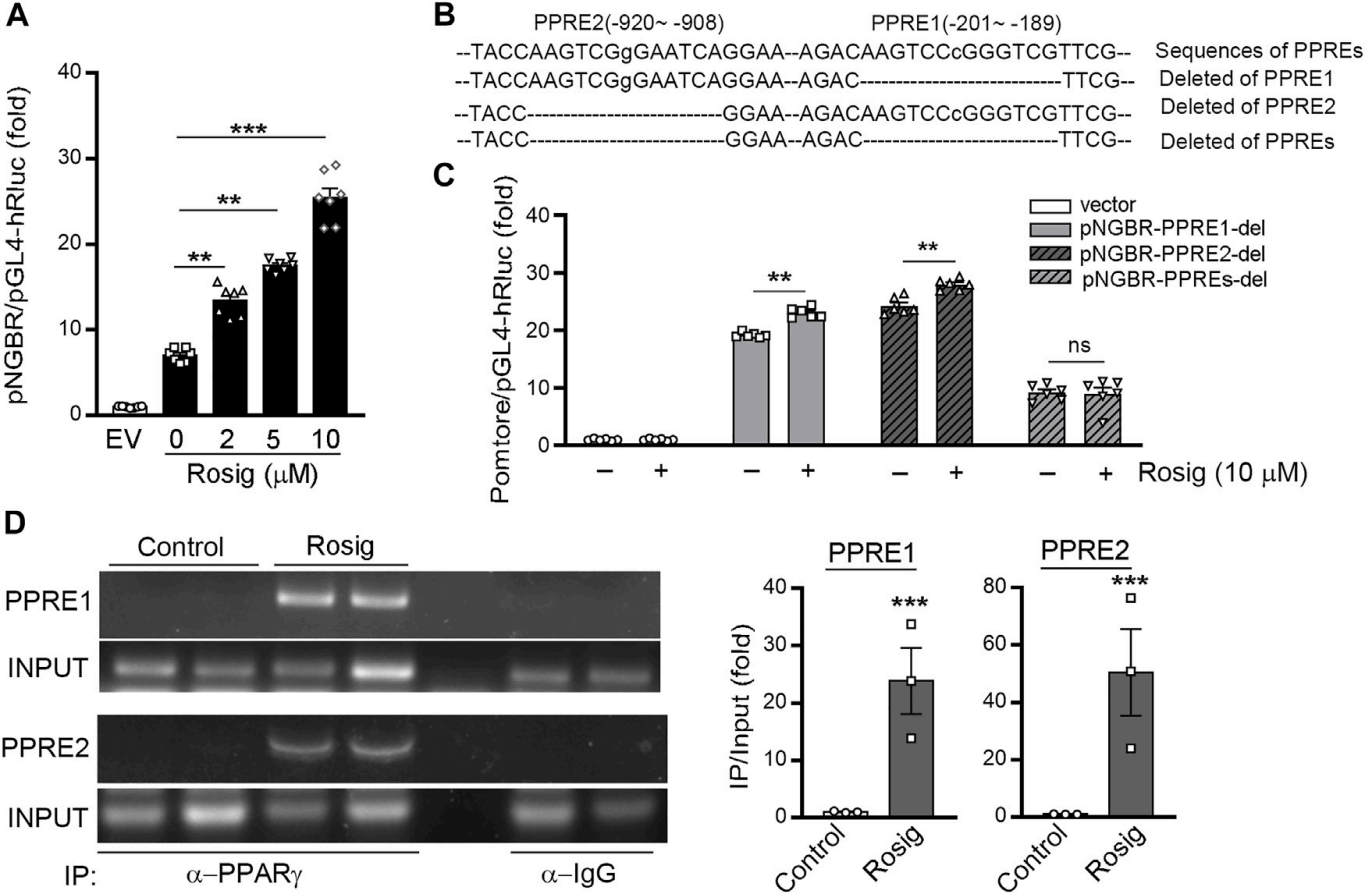
PPARγ activates NGBR transcription. **(A)** 293T cells were transfected with pNGBR (from −983 to +167) and Renila luciferase vector (20:1) overnight, then treated with rosiglitazone at the indicated concentrations. *p < 0.05 (*n* = 7); **(B)** the sequence of NGBR promoter with the two potential PPREs (PPRE1, from −201 to −189; PPRE2, from −920 to −908), individual or both deletion; **(C)** 293T cells were transfected with pGL4 vector, pNGBR-PPRE1-del, pNGBR-PPRE2-del or pNGBR-PPREs-del overnight. Then, the cells received the indicated treatment (rosiglitazone, 10 μM) for 24 h in serum-free medium. Cell lysates were used to determine firefly and Renilla luciferases activity. *p < 0.05; **p < 0.01; ***p < 0.001; ns, not significant (*n* = 6); **(D)** HUVECs were treated with or without rosiglitazone (10 μM) for 24 h. Immunoprecipitation (IP) assay was performed with anti-PPARγ antibody or non-specific IgG overnight. The enrichment of PPARγ protein in the PPRE in NGBR promoter was determined by PCR. *p < 0.05; **p < 0.01; ***p < 0.001 (*n* = 3). Values were expressed as means ± SEM.

The above results suggest PPARγ may activate NGBR expression at the transcriptional level. Therefore, we constructed an 1152 bp (from -983 to +167) human NGBR promoter. We observed that rosiglitazone significantly activated NGBR promoter activity, and the promoter activity was gradually increased with increased rosiglitazone concentration (Figure 3A). We discovered two putative PPRE motifs in the NGBR promoter by sequence alignment analysis, which were defined as PPRE1 (from −201 to −189)and PPRE2 (from −920 to −908). To identify which PPRE plays a role in PPARγ-induced NGBR transcription, we constructed human NGBR promoters without PPRE1 or PPRE2 or both PPREs (Figure 3B). The results in Figure 3C showed that rosiglitazone activated NGBR promoter with PPRE1 or PPRE2 deletion, while the activation was blocked with both PPREs deletion, indicating both PPREs were necessary for PPARγ ligand-induced NGBR expression. Furthermore, we determined that rosiglitazone enhanced the binding of PPARγ with both PPREs in NGBR promoter by ChIP assay (Figure 3D). Taken together, the results in Figure 3 demonstrate that NGBR is a PPARγ target gene, and activation of PPARγ induces NGBR expression at the transcriptional level.

### PPARγ Activation Induces NGBR Expression *in vivo*

**FIGURE 4:**
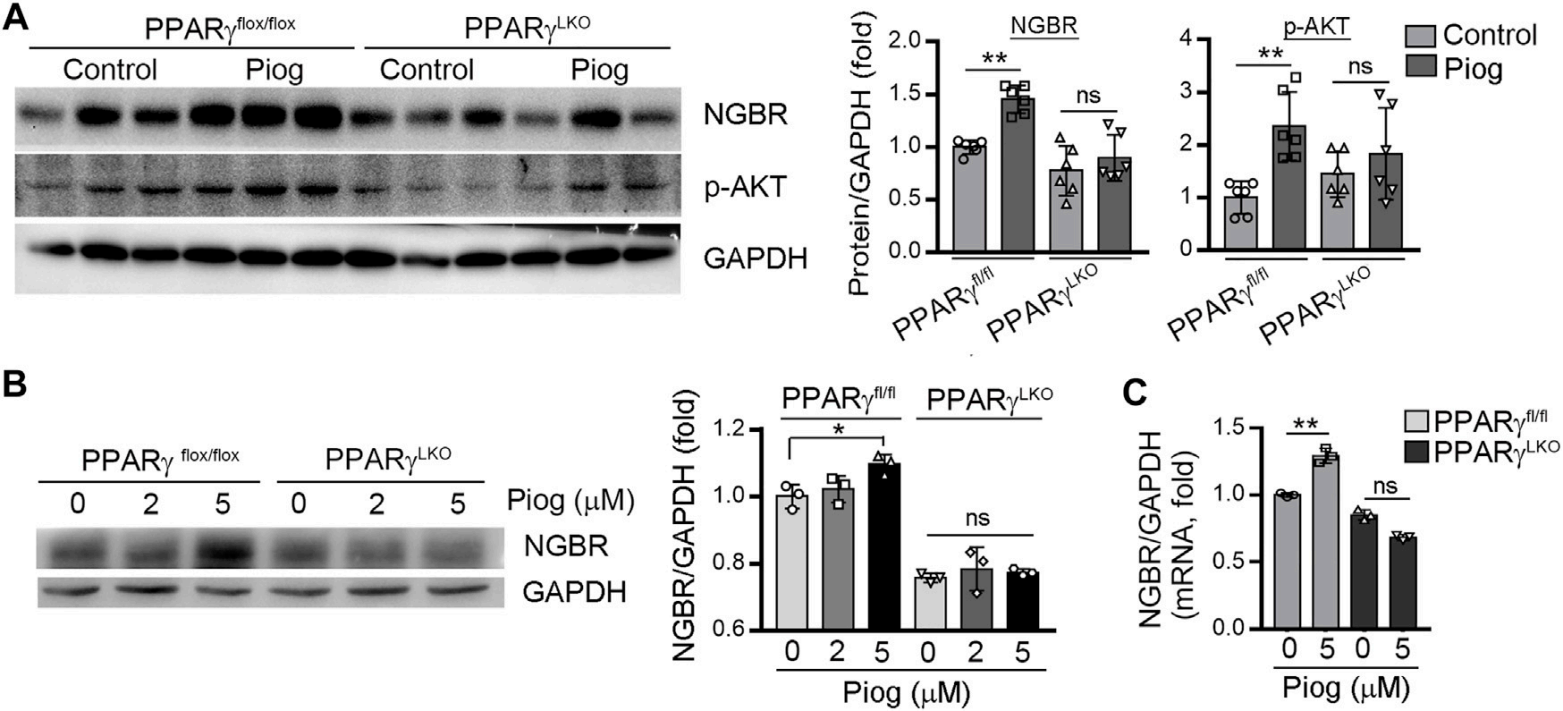
PPARγ plays a significant role in pioglitazone-activated NGBR expression *in vivo*. **(A)** Pioglitazone (30 mg/kg) or corn oil was i.g. administrated with PPARγ^flox/flox^ and PPARγLKO mice for 5 days. After treatment, liver total proteins were collected. Expression of NGBR and *p*-AKT in mouse liver was determined by Western blot. Values were expressed as means ± SD. *p < 0.05; ns, not significant (*n* = 6); **(B, C)** mouse primary hepatocytes treated with vehicle or pioglitazone (10 μM) for 24 h. The expression of NGBR protein and mRNA was determined by Western blot **(B)** or qPCR **(C)**, respectively. Values were expressed as means ± SD. *p < 0.05; **p < 0.01; ns, not significant (*n* = 3).

To determine if activation of PPARγ can enhance NGBR production *in vivo*, PPARγ^fl/fl^ and PPARγ^LKO^ mice were treated with pioglitazone for 5 days. Then the liver was collected to determine NGBR expression. In the liver of PPARγ^fl/fl^ mice pioglitazone increased NGBR expression, which was associated with increased AKT phosphorylation. In contrast, pioglitazone had little effect on hepatic NGBR expression and AKT phosphorylation in PPARγ^LKO^ mice (Figure 4A). At the same time, by isolating mouse primary hepatocytes and treating them with pioglitazone, we found NGBR protein and mRNA expression were increased with pioglitazone in primary hepatocytes sourced from PPARγ^fl/fl^ mice (Figures 4B,C).

### PPARγ Attenuates ER Stress and Inflammation via Induction of NGBR Expression

ER stress and inflammation are observed in various pathologic situations. Our lab previously demonstrated that the knockdown of NGBR in HepG2 cells increased ER stress (Chen et al., 2021). PPARγ knockout (PPARγ^−/-^) mice are developed spontaneous chronic inflammation in lung (Malur et al., 2009). Therefore, we hypothesized that PPARγ-alleviated ER stress and inflammation may be related to its induction of NGBR expression.

**FIGURE 5:**
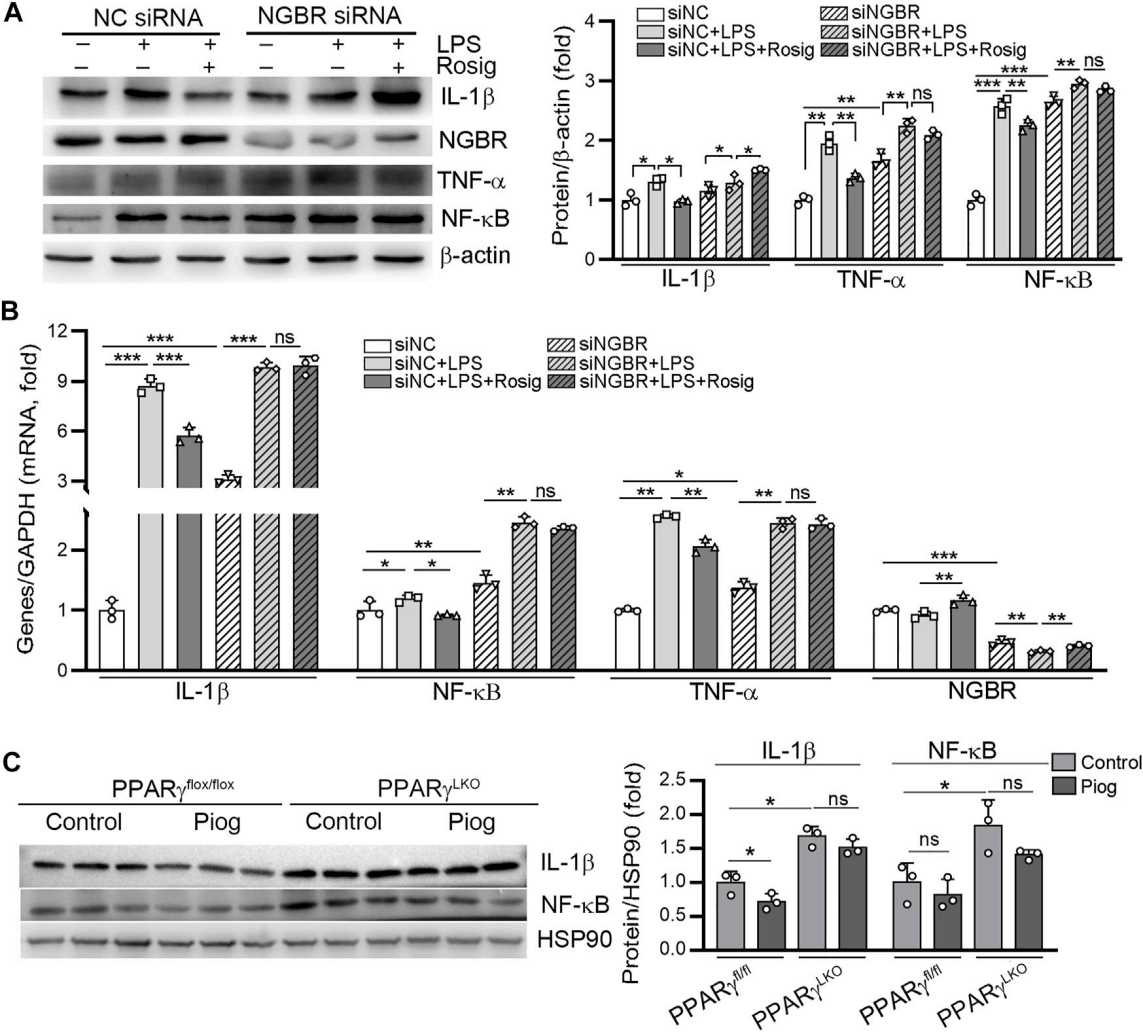
PPARγ attenuates LPS-induced inflammation by regulating NGBR. **(A, B)** HUVEC in a six-well plate were transfected with control siRNA (NC siRNA) or NGBR siRNA for 24 h in serum-free medium, followed by switching the cells into complete medium to culture for another 24 h. After treatment with rosiglitazone (10 μM) for 12 h, all the cells were added with LPS (100 ng/ml) in the presence or absence rosiglitazone for another 12 h. Expression of IL-1β, TNF-α, NF-κB and NGBR protein and mRNA was determined by Western blot **(A)** or qPCR **(B)**, respectively. Values were expressed as means ± SD. *p < 0.05; **p < 0.01; ns, not significant (*n* = 3). **(C)** liver total proteins were collected from Figure 4. Expression of IL-1β and NF-κB in mouse liver was determined by Western blot. Values were expressed as means ± SD. *p < 0.05; ns, not significant (*n* = 3).

To assess the involvement of NGBR on PPARγ-reduced inflammation triggered by LPS, we transfected HUVEC with NGBR siRNA. Knockdown of NGBR expression by siRNA increased basal level of IL-1β, TNF-α and NF-κB (Figures 5A,B). However, the inhibitory effects of rosiglitazone on LPS-induced IL-1β, TNF-α and NF-κB expression were abolished in NGBR siRNA-transfected cells. Interestingly, we determined that IL-1β and NF-κB expression were markedly increased in liver of PPARγ LKO mice. The expression of IL-1β and NF-κB reduced by pioglitazone was diminished in PPARγ^LKO^ mice (Figure 5C). These results indicate that the reduction of inflammation by PPARγ was related to NGBR induction.

**FIGURE 6:**
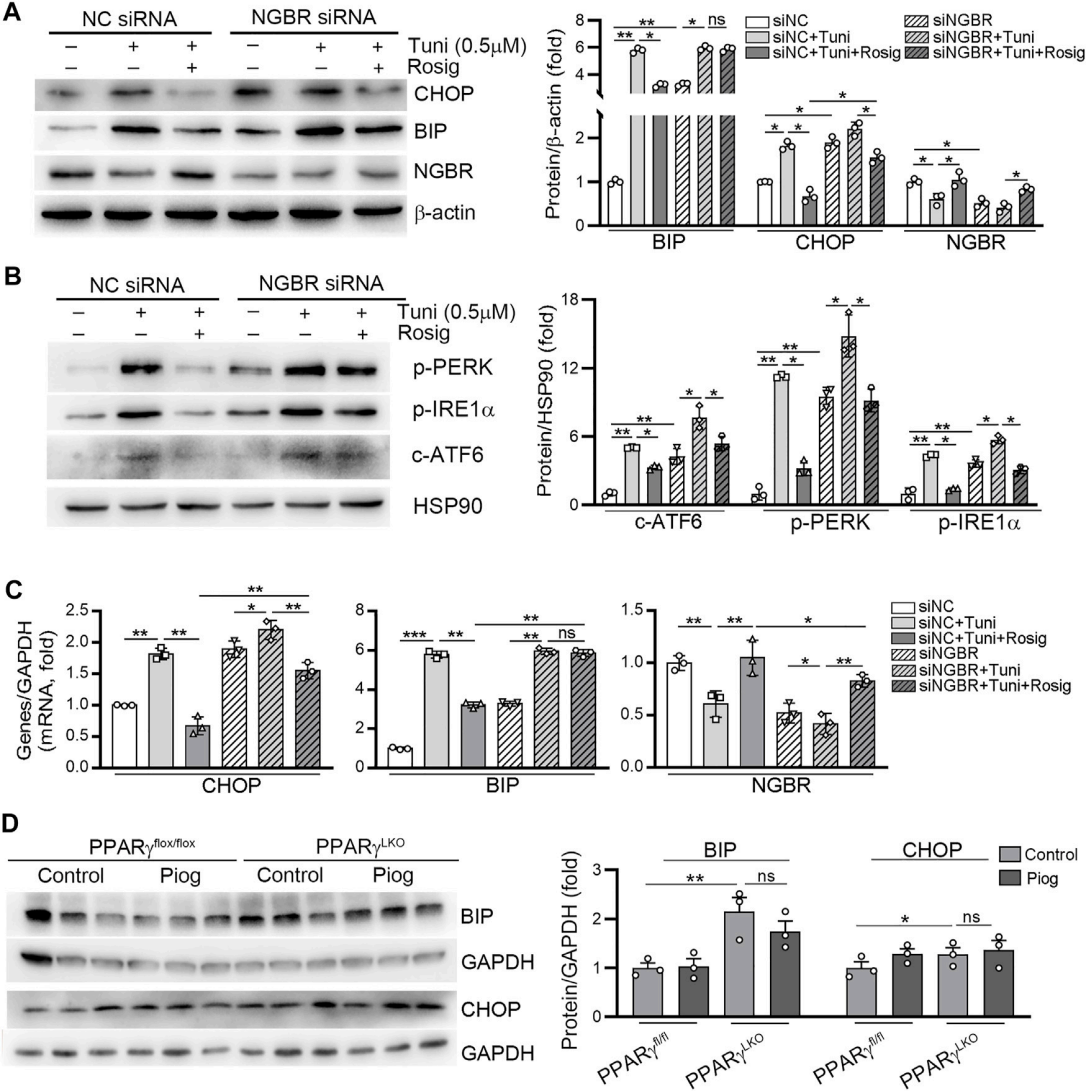
PPARγ reduces tunicamycin-induced ER stress by regulating NGBR. **(A–C)** HUVEC cells in a six-well plate were transfected with control siRNA (NC siRNA) or NGBR siRNA for 24 h in serum-free medium, followed by switching the cells into complete medium to culture for another 24 h. After treatment with rosiglitazone (10 μM) for 12 h, the cells were treated with tunicamycin (0.5 μg/ml) with or without rosiglitazone for another 12 h. Expression of CHOP, BIP, NGBR p-PERK, p-IRE1α and c-ATF6 protein was determined by Western blot **(A, B)**. Expression of CHOP, BIP and NGBR mRNA was determined by qPCR **(C)**. Values were expressed as means ± SD. *p < 0.05; **p < 0.01; ns, not significant (*n* = 3). **(D)** liver total proteins were collected from Figure 4. Expression of BIP and CHOP in mouse liver was determined by Western blot. Values were expressed as means ± SD. *p < 0.05; **p < 0.01; ns, not significant (*n* = 3).

Similar to our previous study, knockdown of NGBR in HUVECs increased BIP and CHOP expression. Meanwhile, the inhibitory effects of rosiglitazone on tunicamycin-induced BIP protein and mRNA expression were abolished in NGBR siRNA-transfected cells (left panel, Figure 6A; middle panel, Figure 6C). In comparison, rosiglitazone-reduced CHOP expression was significantly attenuated in NGBR knockdown cells (middle panel, Figure 6A; left panel, Figure 6C). Moreover, we determined that the phosphorylation levels of PERK and IRE1α (p-PERK, p-IRE1α) as well as the cleavage of ATF6 (c-ATF6) were increased in NGBR knockdown cells (Figure 6B). Meanwhile, the inhibitory effects of rosiglitazone on tunicamycin-induced p-PERK, p-IRE1α and c-ATF6 were significantly attenuated in NGBR siRNA-transfected cells (Figure 6B). *In vivo*, the expression of BIP and CHOP was increased in the liver of PPARγ^LKO^ mice. In PPARγ^fl/fl^ mice, pioglitazone had little effect on BIP and CHOP expression which could be attributed to the low expression of those proteins in the basal level (Figure 6D). Interestingly, pioglitazone had no effect on BIP and CHOP expression in PPARγ^LKO^ mice (Figure 6D). Taken together, these results suggest that rosiglitazone can reduce ER stress in HUVEC and mouse liver by activating PPARγ-NGBR pathway.

## Discussion

NGBR has been proved involved in many pathophysiological processes. For instance, the transduction of adenovirus NGBR can reduce free cholesterol levels and increase NPC2 levels. Mechanistic data demonstrated that the critical role of NGBR in modulating cholesterol trafficking via binding to NPC2 (Harrison et al., 2009). Interestingly, the liver-specific loss of NGBR led to increased FFA and TG levels, which was related to the activation of liver X receptor (LXR) in an NPC2-independent manner (Hu et al., 2016). We previously found that statins reduced oxysterol production and activated NGBR expression, which worked cooperatively to inactivate LXRα and restrict NAFLD (Zhang et al., 2018).

The regulation of NGBR expression and the involved mechanisms have not been fully clarified. Firstly, we observed PPARγ activation-induced NGBR expression in HUVEC, HepG2 cells, and mouse primary hepatocytes (Figures 1, 2). Rosiglitazone-stimulated expression of NGBR was abolished by GW9662 (Figures 1E,F). Then, we found two PPREs in the NGBR promoter region (Figure 3B). We disclosed that PPARγ activated NGBR expression at the transcriptional level by promoter assay and CHIP assay (Figure 3). Finally, we found that lacking PPARγ expression in mouse liver abolished rosiglitazone-induced NGBR expression by using PPARγ^LKO^ mice (Figure 4). Functionally, we found that PPARγ-induced NGBR expression may be one of the underlying mechanisms by which PPARγ activation attenuated ER stress and inflammation.

Thiazolidinediones (TZDs) are insulin sensitizers and effective agonists of PPARγ used against T2D to increase insulin sensitivity. Insulin resistance and endothelial dysfunction are fundamental features of most patients with T2D (Steinberg et al., 1996; Ross, 1999; Tontonoz and Spiegelman, 2008). T2D is associated with elevated plasma FFA and the inappropriate deposition of lipids in the liver and skeletal muscle other than fat (Tontonoz and Spiegelman, 2008). FFAs are closely associated with impaired vascular reactivity, a measure of endothelial dysfunction. A study has showed that skeletal muscle insulin resistance may be caused by impaired insulin signaling pathways in endothelial cells (Kubota et al., 2011). Therefore, the accumulation of FFA and TG in muscle and vascular cells is closely related to insulin resistance and compromises systemic glucose disposal (Tontonoz and Spiegelman, 2008). Rosiglitazone improves systemic insulin sensitivity by activating AMPK and enhancing skeletal muscle glucose uptake (Fryer et al., 2002). In this regard, we previously determined that liver NGBR specific knockout was associated with insulin resistance and loss of β-cells in pancreas (Chen et al., 2021). Conversely, overexpression of NGBR in the liver improved insulin sensitivity by activating AMPKα and insulin signaling pathways (Chen et al., 2021). In the current study, we found PPARγ activation increased NGBR expression, which was related to enhanced AKT phosphorylation (Figures 2, 4A), indicating the PPARγ-enhanced insulin sensitivity may relate to its induction of NGBR expression.

ER stress interplays with many different inflammatory and stress signaling pathways to interfere with insulin signaling (Hotamisligil, 2010). The inflammatory cytokines, such as interleukin-1β (IL-1β) which is primarily driven by NF-κB and/or NLRP3 inflammasome activation, were related to islet inflammation and insulin secretion as well as the development of insulin resistance (Boni-Schnetzler and Donath, 2011). Interestingly, NF-κB and/or NLRP3 inflammasome can be activated directly by ER stress in cultured islets (Hotamisligil, 2010; Lerner et al., 2012). Many studies have shown PPARγ agonists reduce inflammation in multiple immune cells (Bessueille and Magne, 2015). For instance, PPARγ regulates macrophage polarization toward an anti-inflammatory phenotype (M2) (Duan et al., 2009). The differentiation of M2 macrophages results in increased expression of PPARγ. In contrast, PPARγ agonists inhibit the M1 phenotype and decrease the expression of inflammatory cytokines, such as TNF-α, IL-1β, and IL-6 (Berry et al., 2007). Moreover, PPARγ regulates the inflammatory involvement of the immunogenicity of dendritic cells observed in atherosclerosis (Nencioni et al., 2002). Mechanistically, PPARγ represses the transcriptional activation of inflammatory response genes via SUMOylation of the PPARγ ligand-binding domain. SuMOylated PPARγ stabilized nuclear receptor corepressor–histone deacetylase-3 complexes within the promoter region of most inflammatory genes, which represses those genes expression (Pascual et al., 2005). Herein, we determined that PPARγ ligand rosiglitazone reduced LPS-induced inflammation in control HUVECs, which was abolished in NGBR siRNA transfected cells (Figure 5), suggesting that NGBR is an important mediator involved in PPARγ-inhibited inflammation.

In conclusion, our study shows that NGBR is a new molecular target of PPARγ and demonstrates that PPARγ attenuates ER stress and inflammation partly through the induction of NGBR expression. Our study also suggests that PPARγ activation may enhance insulin sensitivity via regulating NGBR expression.

## Data Availability

The original contributions presented in the study are included in the article/supplementary material, further inquiries can be directed to the corresponding author.

## Abbreviations

AKT, Protein kinase B; AMPK, adenosine monophosphate activated protein kinase; ATF6, activating transcription factor 6; BIP, binding immunoglobulin protein; Chop, C/EBP homologous protein; ER stress, endoplasmic reticulum stress; LPS, Lipopolysaccharide; NGBR, Nogo-B receptor; NF-κB, nuclear factor κB; PPARγ, Proliferator-activated receptor gamma; p-IRE1α, phosphorylated inositol-requiring enzyme 1α; p-PERK, phosphorylated protein kinase RNA-dependent ER kinase; TNF-α, tumor necrosis factor alpha; IL-1β, interleukin 1beta.
